# Supplementary figures and images for: An essential role for an Fe-S cluster protein in the cytochrome c oxidase complex of Toxoplasma parasites
Source: PLoS Pathog. 2023 Jun 1;19(6):e1011430. doi: 10.1371/journal.ppat.1011430 (PMC10263302; doi:10.1371/journal.ppat.1011430)

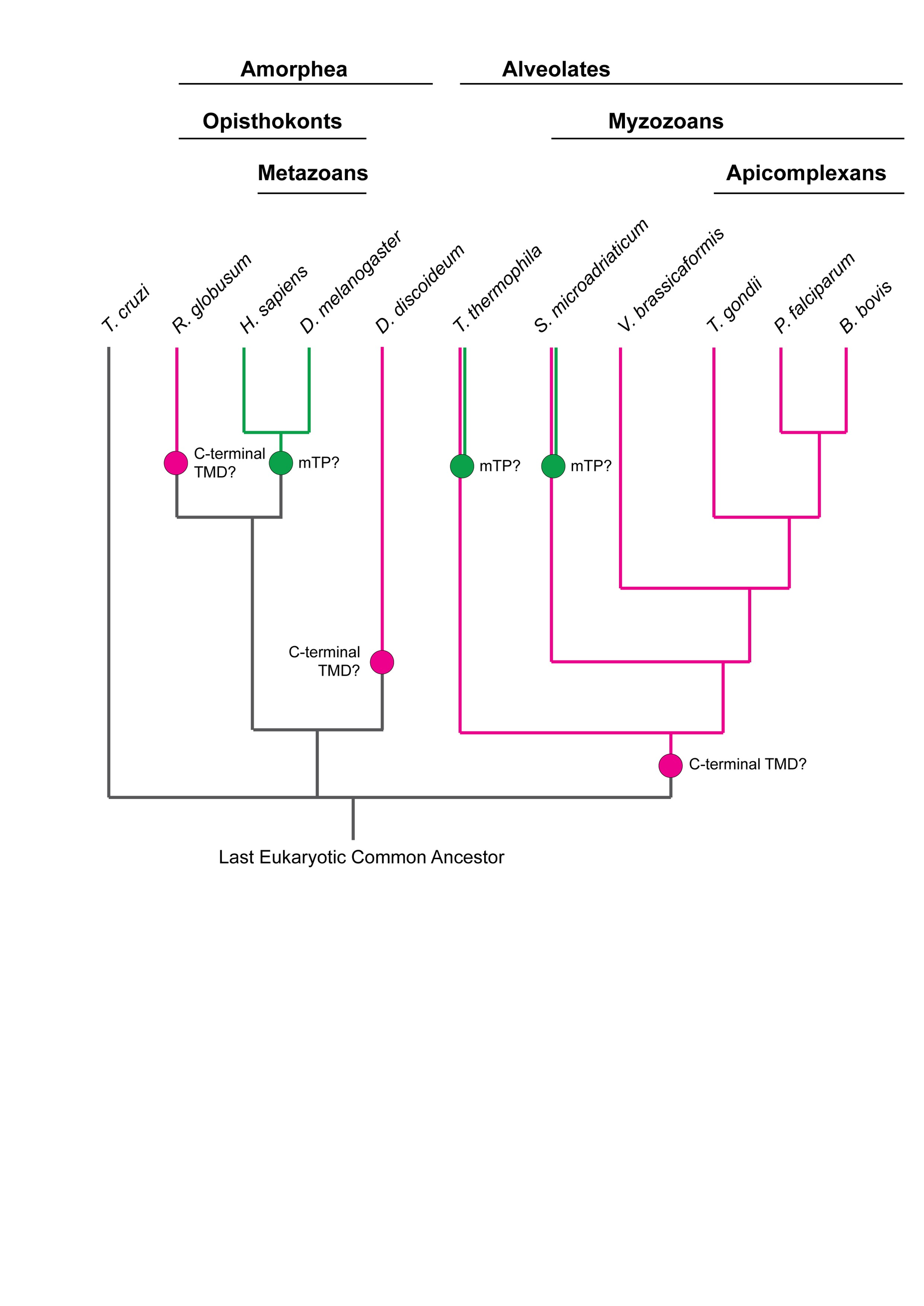

Supplement: S1 Fig — An illustrative phylogenetic tree based on currently accepted models for eukaryotic evolution [54]. Branches were coloured based on whether the ApiCox13 homologs of clade members contain a C-terminal transmembrane domain (TMD, pink) and/or an N-terminal mitochondrial targeting peptide (mTP, green) (S1 Table). Possible points at which TMD or mTP features were gained are indicated by coloured circles. (TIF) [file ppat.1011430.s001.tif]

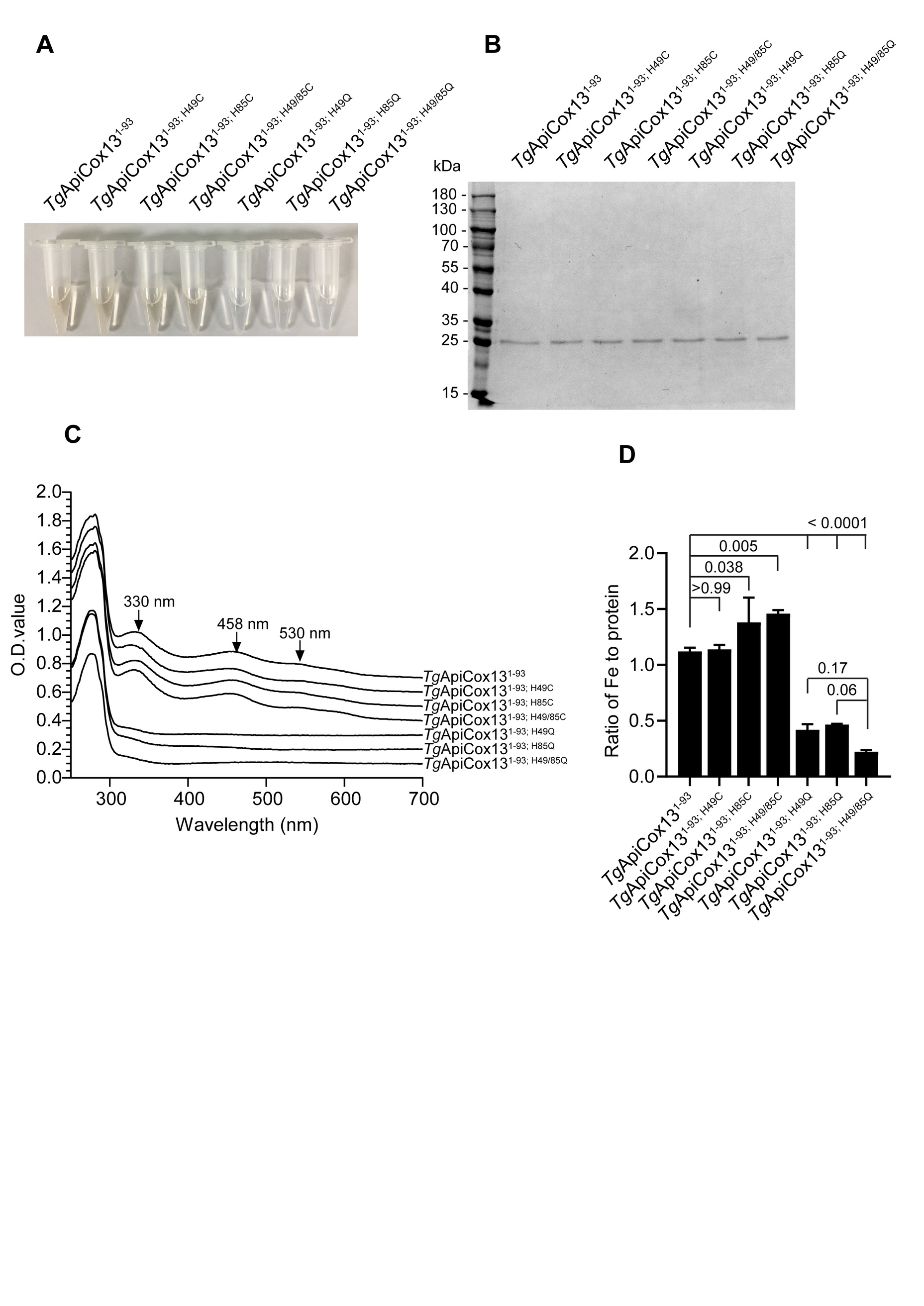

Supplement: S2 Fig — Purified recombinant TgApiCox131-93 and the Fe-S cluster binding site mutants TgApiCox131-93; H49C, TgApiCox131-93; H85C, TgApiCox131-93; H49/85C, TgApiCox131-93; H49Q, TgApiCox131-93; H85Q and TgApiCox131-93; H49/85Q were analysed for (A) colour of samples (red vs clear), (B) molecular mass by SDS-PAGE stained with Coomassie, and for Fe-S cluster binding by (C) UV-visible absorption spectroscopy and (D) iron content measurements given as a ratio of Fe to protein in mol. Iron content measures represent the mean ± standard deviation from n = 3 independent experiments. One way ANOVA and a Tukey’s multiple comparisons test was performed, with relevant p-values shown. (TIF) [file ppat.1011430.s002.tif]

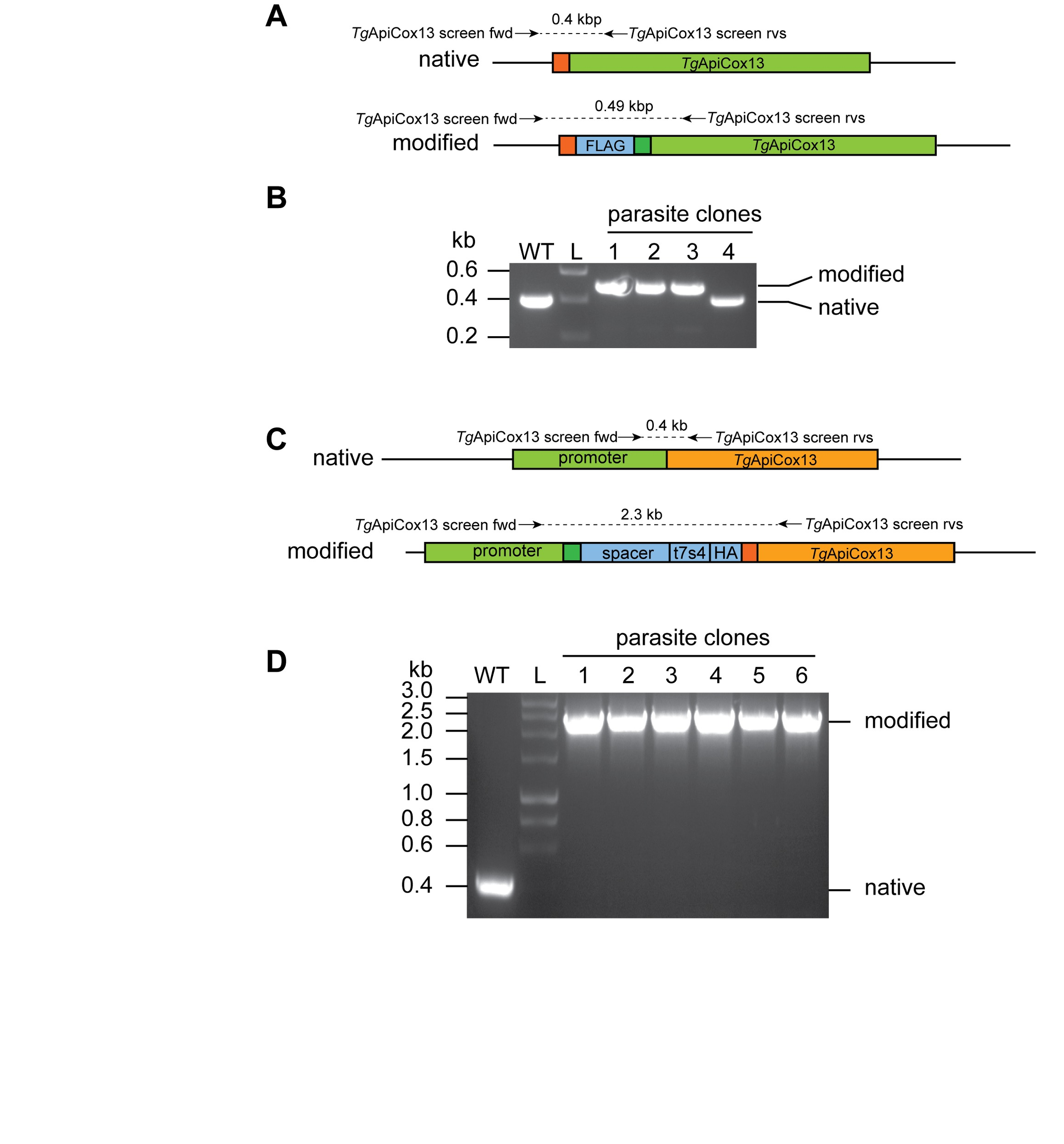

Supplement: S3 Fig — (A) Diagram depicting the 5’ replacement strategy to introduce a FLAG epitope tag into the TgApiCox13 locus of rTgApiCox25-HA parasites. A sgRNA was designed to target the T. gondii genome near the start codon of TgApiCox13. A plasmid containing the sgRNA and GFP-tagged Cas9 endonuclease was co-transfected into T. gondii parasites with a PCR product encoding a FLAG epitope tag flanked by 50 bp of sequence homologous to the regions immediately up- and down-stream of the TgApiCox13 start codon. Forward and reverse primers (TgApiCox13 screen fwd and rvs) were designed to screen parasite clones for integration of the FLAG tag at the TgApiCox13 locus, yielding a 400 bp product in the native locus and a 490 bp product in the modified locus. (B) PCR screening using genomic DNA extracted from putative FLAG-TgApiCox13 parasites (clones 1–4). Clones 1–3 yielded PCR products that indicated they had been successfully modified. Genomic DNA extracted from wild type (WT) parasites was used as a control. (C) Diagram depicting the 5’ replacement strategy to simultaneously HA-tag and replace the native promoter of TgApiCox13 to generate ATc-regulated HA-TgApiCox13 parasites. The same sgRNA described in (A) was used to target the T. gondii genome near the start codon of TgApiCox13. A plasmid containing the sgRNA and GFP-tagged Cas9 endonuclease was co-transfected into T. gondii parasites with a PCR product encoding the ATc regulatable ‘t7s4’ promoter, which contains 7 copies of the Tet operon and a Sag4 minimal promoter, plus a start codon immediately followed by a HA epitope tag, flanked by 50 bp of sequence homologous to the regions immediately up- and down-stream of the TgApiCox13 start codon. The PCR product also contains a ‘spacer’ region that separates the regulatable promoter from the native promoter of the TgApiCox13 gene to enable sufficient regulation. The same primers described in (A) were used to screen parasite clones for integration of the regulatable promoter and HA tag [file ppat.1011430.s003.tif]

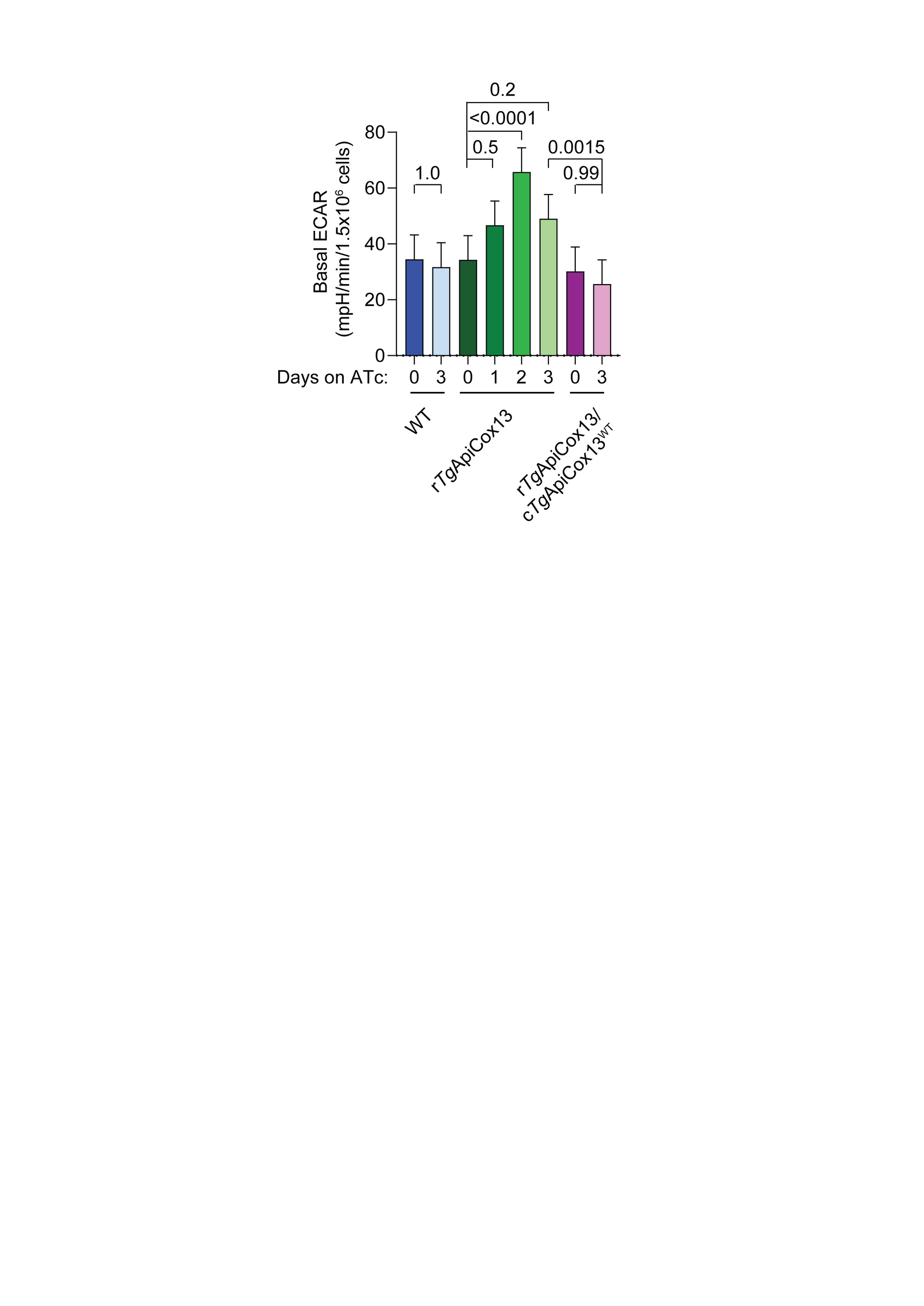

Supplement: S4 Fig — Basal extracellular acidification rate (ECAR) of WT (blue), rHA-TgApiCox13 parasites (green) or rHA-TgApiCox13/cFLAG-TgApiCox13WT (purple) parasites grown in the absence of ATc or in the presence of ATc for 1–3 days. A linear mixed-effects model was fitted to the data and values depict the estimated marginal mean ± 95% CI of three independent experiments. ANOVA followed by Tukey’s multiple pairwise comparisons test was performed, with relevant p values shown. (TIF) [file ppat.1011430.s004.tif]

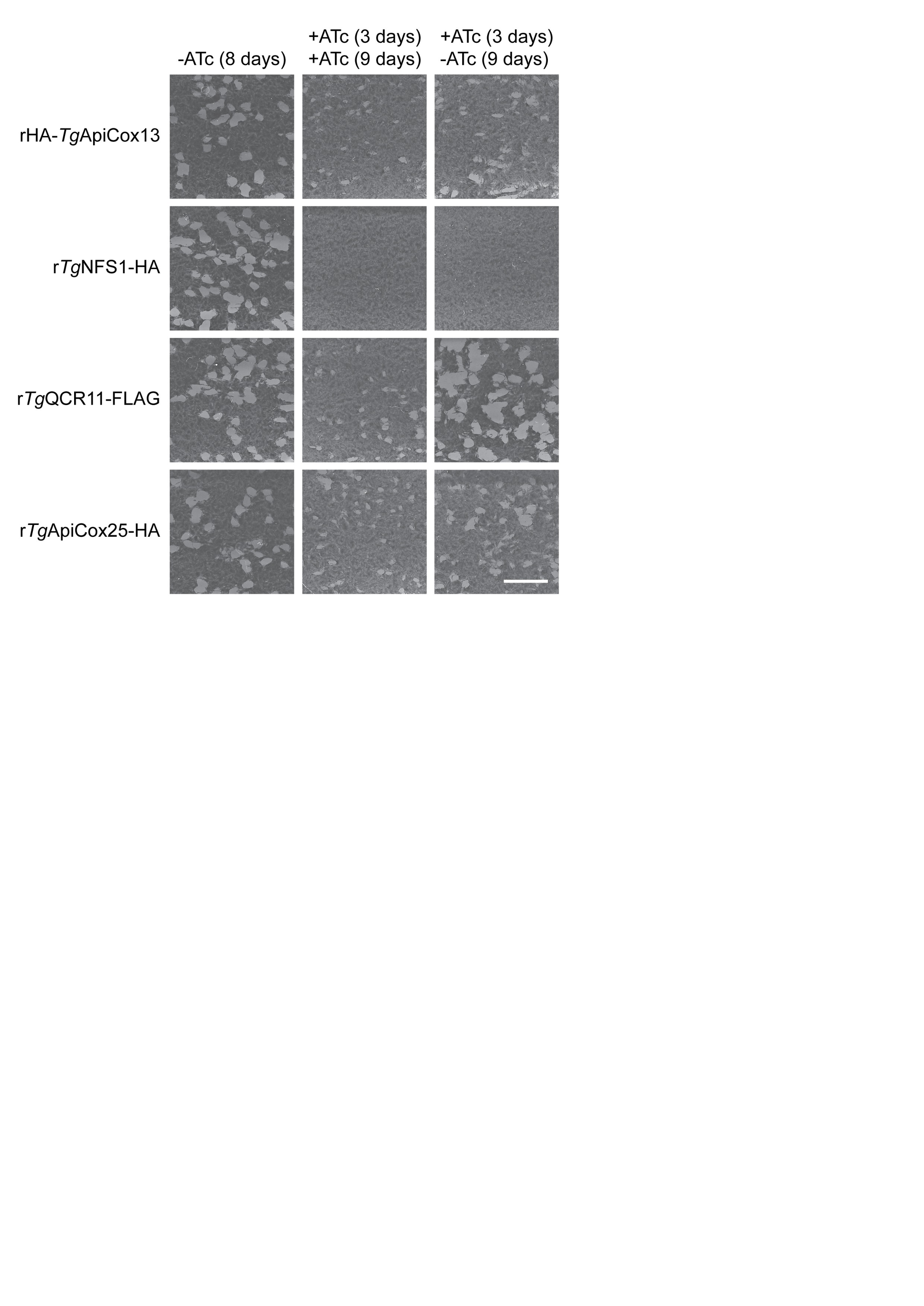

Supplement: S5 Fig — Plaque assays of rHA-TgApiCox13 (top), rTgNFS1-HA (upper middle), rTgQCR11-FLAG (lower middle) and rTgApiCox25-HA (bottom) parasites grown in the absence of ATc for 8 days (left), grown in the presence of ATc for all 12 days (middle) or pre-incubated with ATc for 3 days before washing out the ATc and growing for a further 9 days in the absence of ATc (right). Plaque assays are from a single experiment and are representative of four independent experiments. Scale bar represents 1 cm. (TIF) [file ppat.1011430.s005.tif]

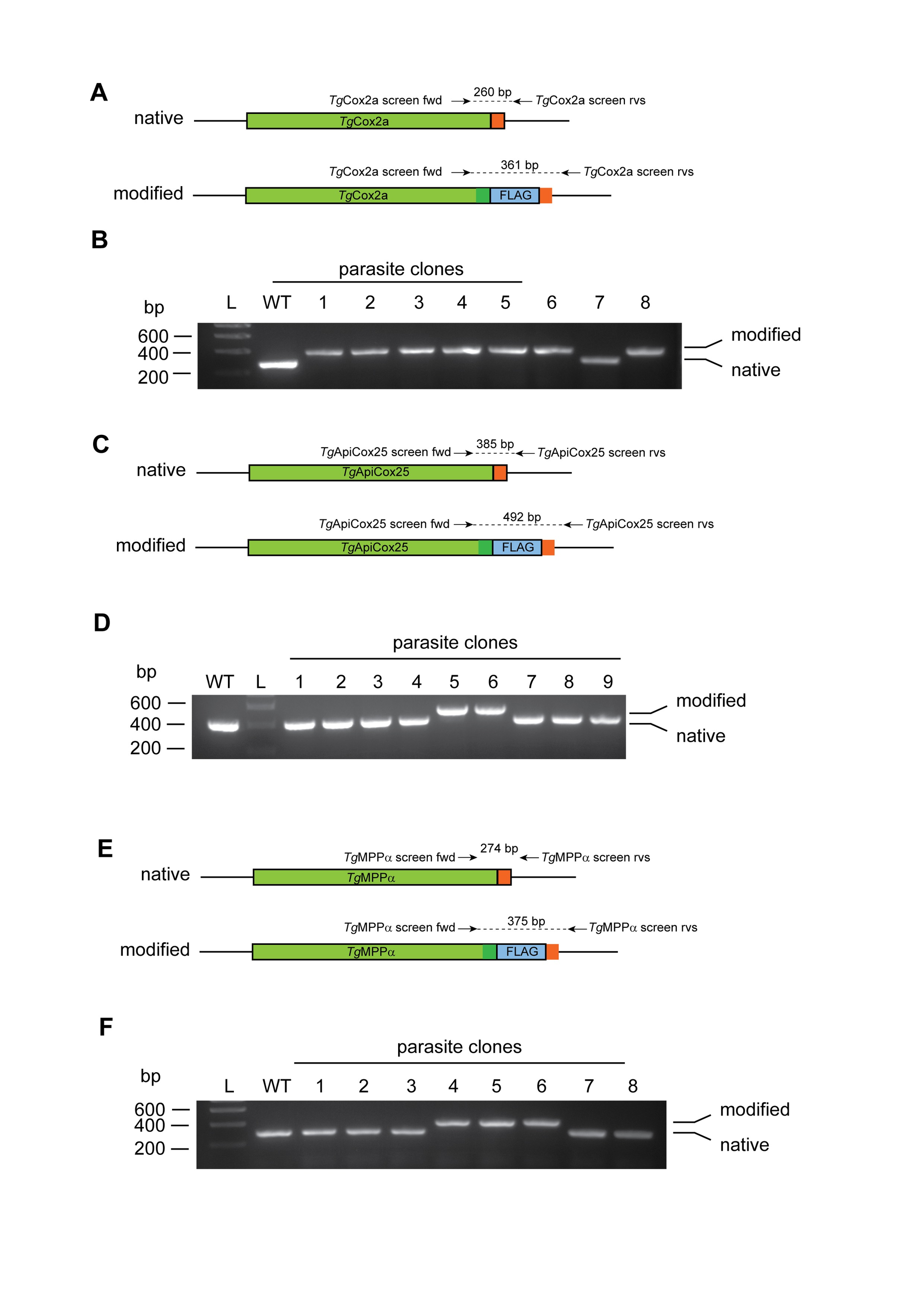

Supplement: S6 Fig — Diagrams depict the 3’ replacement strategy to FLAG-tag target genes. sgRNAs were designed to target the T. gondii genome near the stop codon of target genes. A plasmid containing the sgRNA and GFP-tagged Cas9 endonuclease was co-transfected into rHA-TgApiCox13 T. gondii parasites with a PCR product encoding a FLAG epitope tag flanked by 50 bp of sequence homologous to the regions immediately up- and down-stream of the stop codon. Genomic DNA extracted from wild type (WT) parasites was used as a control in PCRs. (A) Forward and reverse primers were designed to screen parasite clones for integration of the FLAG tag at the TgCox2a locus, yielding a 260 bp product in the native locus and a 361 bp product in the modified locus. (B) PCR screening using genomic DNA extracted from putative TgCox2a-FLAG parasites (clones 1–8). Clones 1–6 and 8 yielded PCR products that indicated they had been successfully modified. (C) Forward and reverse primers were designed to screen parasite clones for integration of the FLAG tag at the TgApiCox25 locus, yielding a 385 bp product in the native locus and a 492 bp product in the modified locus. (D) PCR screening using genomic DNA extracted from putative TgApiCox25-FLAG parasites (clones 1–9). Clones 5 and 6 yielded PCR products that indicated they had been successfully modified. (E) Forward and reverse primers were designed to screen parasite clones for integration of the FLAG tag at the TgMPPα locus, yielding a 274 bp product in the native locus and a 375 bp product in the modified locus. (F) PCR screening using genomic DNA extracted from putative TgMPPα-FLAG parasites (clones 1–8). Clones 4–6 yielded PCR products that indicated they had been successfully modified. (TIF) [file ppat.1011430.s006.tif]

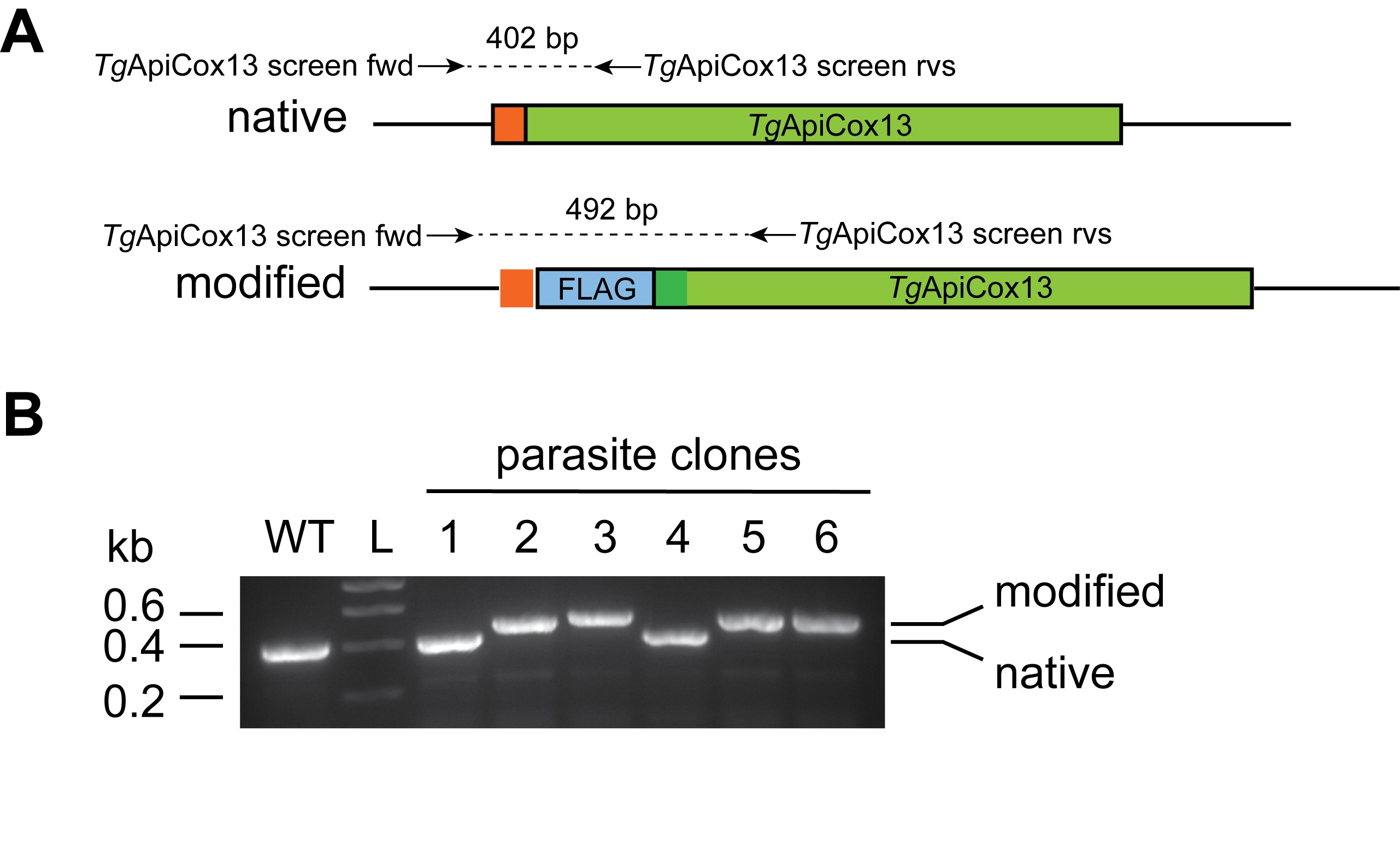

Supplement: S7 Fig — (A) Diagram depicting the 5’ replacement strategy to FLAG-tag TgApiCox13. The same plasmid described in S3A Fig containing the sgRNA and GFP-tagged Cas9 endonuclease was co-transfected into rHA-TgApiCox13 T. gondii parasites with a PCR product encoding a FLAG epitope tag flanked by 50 bp of sequence homologous to the regions immediately up- and down-stream of the TgApiCox13 start codon. Forward and reverse primers were designed to screen parasite clones for integration of the FLAG tag at the TgApiCox13 locus, yielding a 402 bp product in the native locus and a 492 bp product in the modified locus. (B) PCR screening using genomic DNA extracted from putative FLAG-TgApiCox13 parasites (clones 1–6). Clones 3, 5 and 6 yielded PCR products that indicated they had been successfully modified. Genomic DNA extracted from wild type (WT) parasites was used as a control. (TIF) [file ppat.1011430.s007.tif]

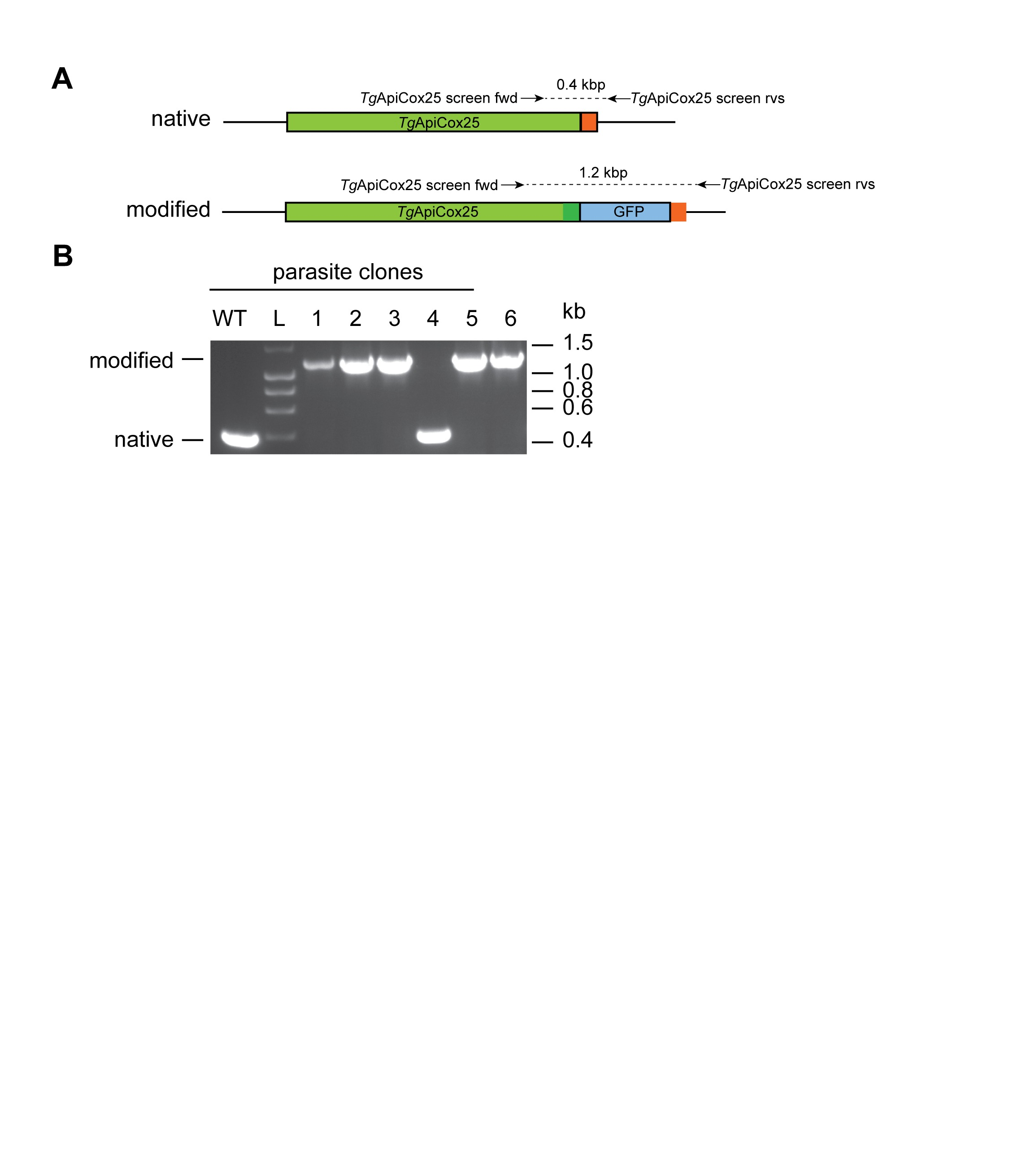

Supplement: S8 Fig — (A) Diagram depicting the 3’ replacement strategy to GFP-tag TgApiCox25. The same plasmid described in S6C Fig containing the sgRNA and GFP-tagged Cas9 endonuclease was co-transfected into rHA-TgApiCox13 T. gondii parasites together with a PCR product encoding a GFP epitope tag flanked by 50 bp of sequence homologous to the regions immediately up- and down-stream of the TgApiCox25 stop codon. Forward and reverse primers were designed to screen parasite clones for integration of the GFP tag at the TgApiCox25 locus, yielding a 400 bp product in the native locus and a 1.2 kbp product in the modified locus. (B) PCR screening using genomic DNA extracted from putative TgApiCox25-GFP parasites (clones 1–6). Clones 1–3 and 5–6 yielded PCR products that indicated they had been successfully modified. (TIF) [file ppat.1011430.s008.tif]

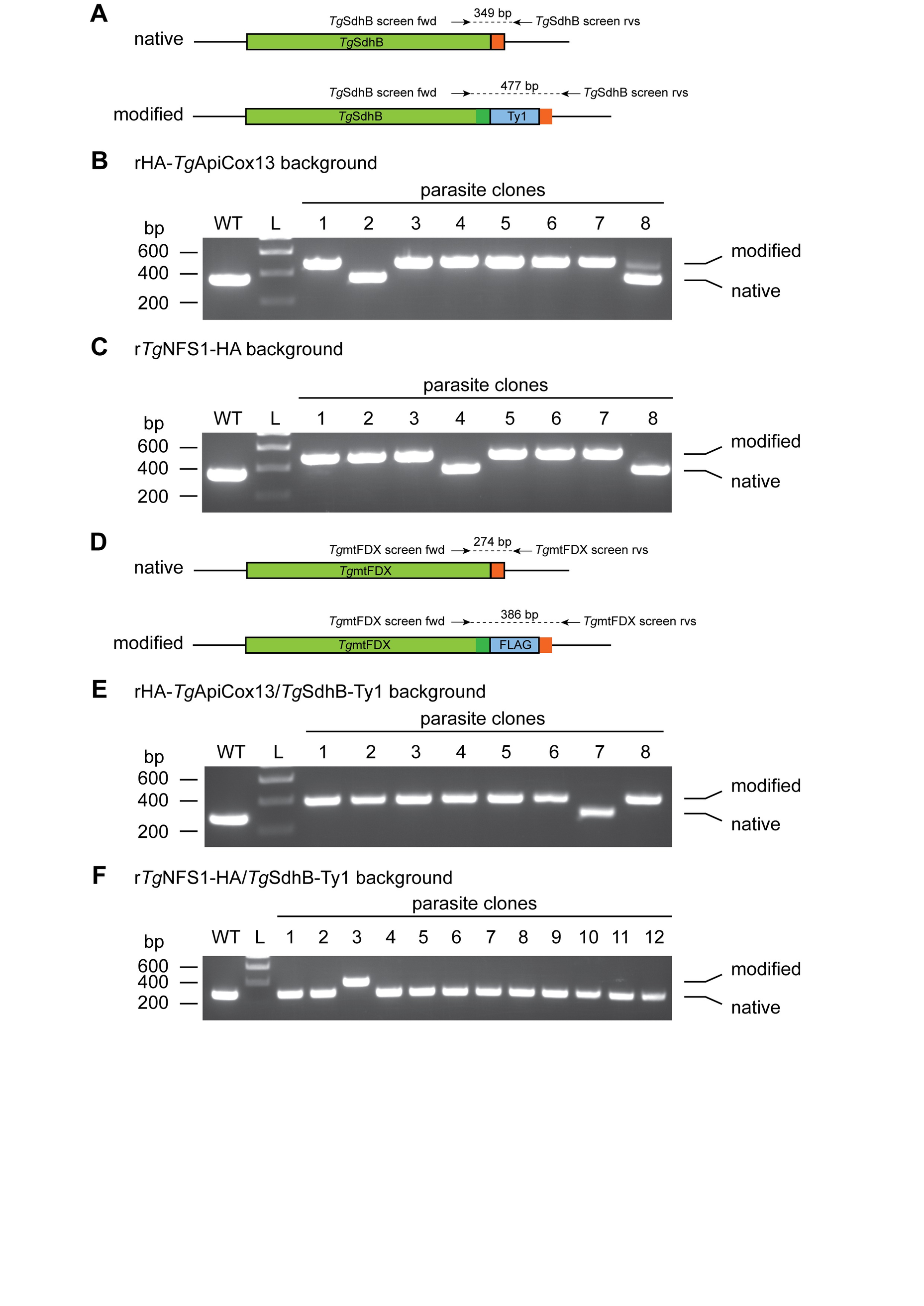

Supplement: S9 Fig — (A) Diagram depicting the 3’ replacement strategy to Ty1-tag TgSdhB. A plasmid containing a sgRNA targeting near the stop codon of the TgSdhB open reading frame and GFP-tagged Cas9 endonuclease was co-transfected into rHA-TgApiCox13 or TgNFS1-HA T. gondii parasites together with a PCR product encoding a Ty1 epitope tag flanked by 50 bp of sequence homologous to the regions immediately up- and down-stream of the TgSdhB stop codon. Forward and reverse primers were designed to screen parasite clones for integration of the Ty1 tag at the TgSdhB locus, yielding a 349 bp product in the native locus and a 477 bp product in the modified locus. (B-C) PCR screening to test for integration of a Ty1 tag into the TgSdhB locus in (B) rHA-TgApiCox13 or (C) TgNFS1-HA parasites using genomic DNA extracted from clonal parasites. Clones 1 and 3–7 in the rHA-TgApiCox13 line (B) and clones 1–3 and 5–7 in the rTgNFS1-HA line (C) yielded PCR products that indicated they had been successfully modified. (D) Diagram depicting the 3’ replacement strategy to FLAG-tag TgmtFDX. A plasmid containing a sgRNA targeting near the stop codon of the TgmtFDX open reading frame and GFP-tagged Cas9 endonuclease was co-transfected into rHA-TgApiCox13/TgSdhB-Ty1 or TgNFS1-HA/TgSdhB-Ty1 parasites together with a PCR product encoding a FLAG epitope tag flanked by 50 bp of sequence homologous to the regions immediately up- and down-stream of the TgmtFDX stop codon. Forward and reverse primers were designed to screen parasite clones for integration of the FLAG tag at the TgmtFDX locus, yielding a 274 bp product in the native locus and a 386 bp product in the modified locus. (E-F) PCR screening to test for integration of a FLAG tag into the TgmtFDX locus of (E) rHA-TgApiCox13/TgSdhB-Ty1 or (F) TgNFS1-HA TgSdhB-Ty1 parasites using genomic DNA extracted from clonal parasites. Clones 1–6 and 8 in the rHA-TgApiCox13/TgSdhB-Ty1 line (E) and clone 3 in the TgNFS1-HA TgSdhB-Ty1 parasite line (F) yielded PCR products th [file ppat.1011430.s009.tif]

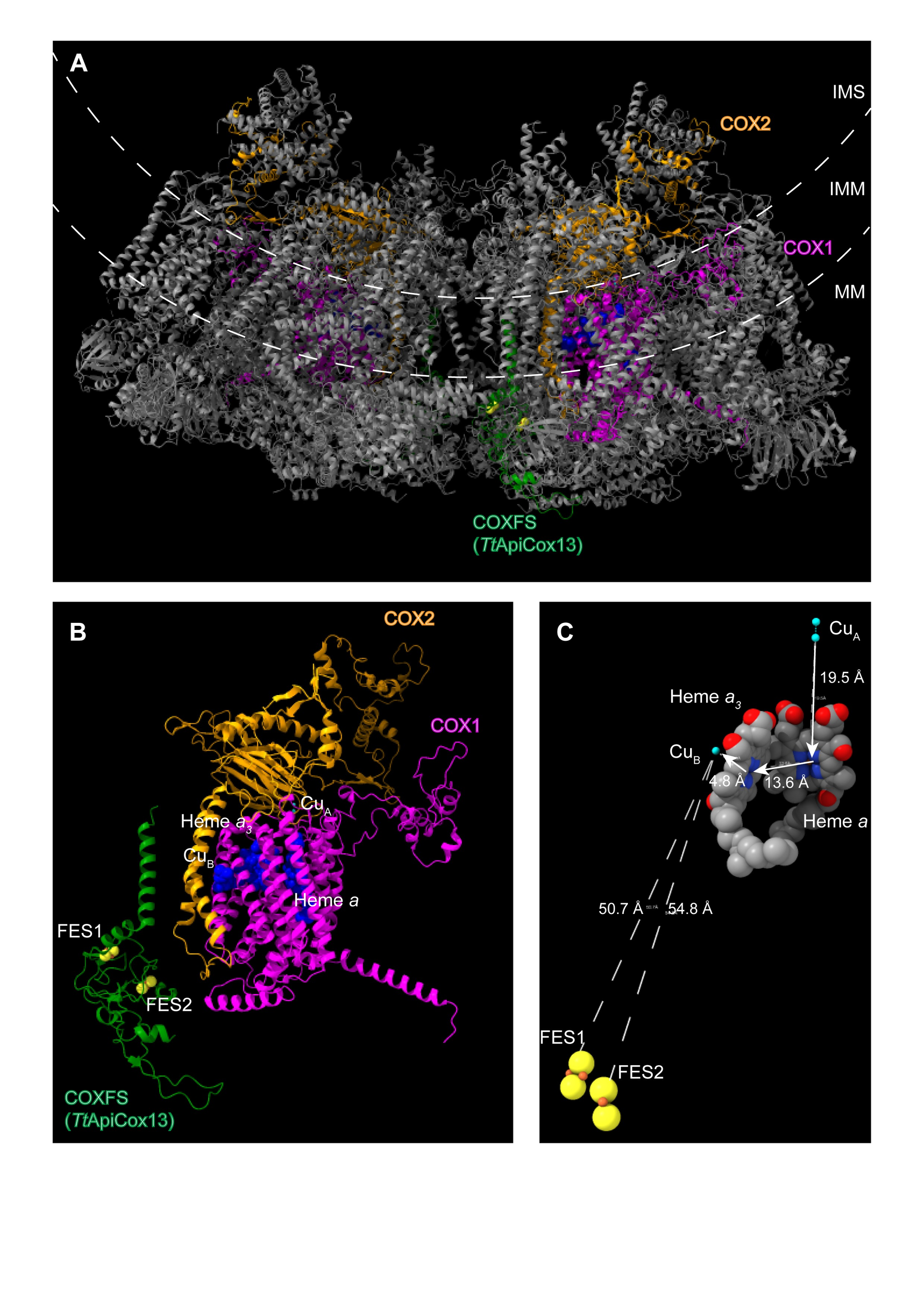

Supplement: S10 Fig — PDB structure 7W5Z [13] was evaluated in Chimera. A) The position of COXFS (green, the T. thermophila homolog of ApiCox13) in the Complex IV dimer relative to the catalytic subunits COX1 (magenta) and COX2 (orange), with all other subunits shown in grey. The heme groups of COX1 are shown in blue, and the Fe-S clusters of COXFS are shown in yellow. The inner mitochondrial membrane (IMM) is depicted by dashed lines with the intermembrane space (IMS) above and the mitochondrial matrix (MM) below. B) The position of COXFS (green) relative to the catalytic subunits COX1 (magenta) and COX2 (orange) without the grey subunits. The functional groups are labelled. C) The path and distance between the electron transferring functional groups within Complex IV are depicted by arrows. The distance between the CuB functional group and the two Fe-S clusters of COXFS are shown by dashed lines. (TIF) [file ppat.1011430.s010.tif]
