# Supplementary material for: An essential role for an Fe-S cluster protein in the cytochrome c oxidase complex of Toxoplasma parasites
Source: PLoS Pathog. 2023 Jun 1;19(6):e1011430. doi: 10.1371/journal.ppat.1011430 (PMC10263302; doi:10.1371/journal.ppat.1011430)
Supplement: S1 Table — We performed BlastP searches of the NCBI database using TgApiCox13 as a query sequence to identify homologs from other organisms, with the protein ID, percent identity to TgApiCox13, and E-value reported. We used the bioinformatics tools MitoProt II to predict presence (+, green) or absence (-, gray) of mitochondrial targeting peptides, and TMHMM to predict presence (+, magenta) or absence (-, gray) of transmembrane domains. Where appropriate, we have also provided the vEuPathDB ID. (PDF) [file ppat.1011430.s011.pdf]

**Organism**	**ID**	**Identity to *Tg*ApiCox13 (%)**	**E-value**	**N-terminal mitochondrial targeting peptide**	**C-terminal transmembrane domain**
Trypanosoma cruzi	KAF8283648.1/ TcBrA4_0059010	48	$3e^{-19}$	-	-
Rhizoclosmatium globusum	ORY44526.1	56	$1e^{-17}$	-	+
Homo sapiens	NP_001129970.1	38	$3e^{-11}$	+	-
Drosophila melanogaster	NP_001260735.1	44	$1e^{-15}$	+	-
Dictyostelium discoideum	XP_647247.1	35	$6e^{-7}$	-	+
Tetrahymena thermophila	XP_001019630.1	46	$2e^{-13}$	+	+
Symbiodinium microadriaticum	CAE7828504.1	46	$5e^{-25}$	+	+
Vitrella brassicaformis	CEL92537.1/ Vbra_4702	60	$3e^{-44}$	-	+
Toxoplasma gondii	TGGT1_254030	100	0	-	+
Plasmodium falciparum	XP_001347506.2/ PF3D7_1022900	55	$8e^{-42}$	-	+
Babesia bovis	XP_001610212.1/ BBOV_II006940	58	$6e^{-47}$	-	+
