# Supplementary material for: An essential role for an Fe-S cluster protein in the cytochrome c oxidase complex of Toxoplasma parasites
Source: PLoS Pathog. 2023 Jun 1;19(6):e1011430. doi: 10.1371/journal.ppat.1011430 (PMC10263302; doi:10.1371/journal.ppat.1011430)
Supplement: S2 Table — (PDF) [file ppat.1011430.s012.pdf]

**Oligonucleotide name**	**Oligonucleotide sequence (5' to 3')**
*Tg*ApiCox13$^{1-93}$ ORF fwd	GAACAGATTGGAGGTCATATGGTGTGGCAAGAGTGG
*Tg*ApiCox13$^{1-93}$ ORF rvs	AGAGTCGACAAGCTTGGATCTTAGTTGTTGTTGCAC
*Tg*ApiCox13$^{1-93;\ H49C}$ fwd	GCCtgCAAAGGCACCCCTTTCAAGCCGGTTATG
*Tg*ApiCox13$^{1-93;\ H49C}$ rvs	AAAGGGGTGCCTTTGcaGGCGCCATCGC
*Tg*ApiCox13$^{1-93;\ H85C}$ fwd	ACTtgCTTGTGGGTGAAGTGCAACAACAAC
*Tg*ApiCox13$^{1-93;\ H85C}$ rvs	CACTTCACCCACAAGcaAGTGCCGTTGCAC
*Tg*ApiCox13$^{1-93;\ H49Q}$ fwd	GCCCAgAAAGGCACCCCTTTCAAGCCGGTTATG
*Tg*ApiCox13$^{1-93;\ H49Q}$ rvs	AAAGGGGTGCCTTTcTGGGCGCCATCGC
*Tg*ApiCox13$^{1-93;\ H85Q}$ fwd	ACTCAgTTGTGGGTGAAGTGCAACAACAAC
*Tg*ApiCox13$^{1-93;\ H85Q}$ rvs	CACTTCACCCACAAcTGAGTGCCGTTGCAC
*Tg*ApiCox13$^{1-93}$ seq fwd	GAGACTCACATCAATTTAAAGGTGTCC
*Tg*ApiCox13$^{1-93}$ seq rvs	GCCAAATGGCAGGGATCTTAG
*Tg*ApiCox13 5' CRISPR fwd	ACGCTTTCTTCACCATGGTGGTTTTAGAGCTAGAAATAGCAAG
Generic CRISPR rvs	ACTTGACATCCCCATTTAC
*Tg*ApiCox13 pro rep fwd	GAGGGTGCTCAGAAGACGAAGGGCTTCATTTGAAAACCTGAAGC<br>GTCTGACGGTTGCAGGCTCCTTCTTCGG
*Tg*ApiCox13 pro rep rvs	GTCGTCTGGGGCTGCGGCTGCGGGTCATACGGCCACCACTCTTG<br>CCACACGCTACCTCCACCGGGTAAGGCATAATCTGG
*Tg*ApiCox13 screen fwd	GATGCGACATCTCCGAATCAC
*Tg*ApiCox13 screen rvs	CTCTCCGGTTCTCTTACCTTCC
N-terminal FLAG gBlock	ACTGCCGTTTGCTTCCCTGCCCTCGTCTTTGCCTTACGCTTTCTTC<br>ACCATGGACTACAAAGACCATGACGGTGATTATAAAGATCATGAC<br>ATCGATTACAAGGATGACGATGACAAGGGTGGAGGTAGCGGTGG<br>TGGAAGTGTGTGGCAAGAGTGGTGGCCGTATGACCCGCAGCCGC<br>AGCCCCAGACGAC
*Tg*ApiCox13 FLAG fwd	ACTGCCGTTTGCTTCCCTG
*Tg*ApiCox13 FLAG rvs	GTCGTCTGGGGCTGCGG
TEV-GFP gBlock	GGTGGAGGTAGCGGTGGTGGAAGTGAAAATCTGTACTTCCAGGG<br>AGGTACCGCGGTGAGCAAGGGCGAGGAGCTGTTCACCGGGGTG<br>GTGCCCATCCTGGTCGAGCTGGACGGCGACGTAAACGGCCACAA<br>GTTCAGCGTGTCCGGCGAGGGCGAGGGCGATGCCACCTACGGC<br>AAGCTGACCCTGAAGTTCATCTGCACCACCGGCAAGCTGCCCGT<br>GCCCTGGCCCACCCTCGTGACCACCCTGACCTACGGCGTGCAGT<br>GCTTCAGCCGCTACCCCGACCACATGAAGCAGCACGACTTCTTCA<br>AGTCCGCCATGCCCGAAGGCTACGTCCAGGAGCGCACCATCTTC<br>TTCAAGGACGACGGCAACTACAAGACCCGCGCCGAGGTGAAGTT<br>CGAGGGCGACACCCTGGTGAACCGCATCGAGCTGAAGGGCATC<br>GACTTCAAGGAGGACGGCAACATCCTGGGGCACAAGCTGGAGTA<br>CAACTACAACAGCCACAACGTCTATATCATGGCCGACAAGCAGAA<br>GAACGGCATCAAGGTGAACTTCAAGATCCGCCACAACATCGAGG<br>ACGGCAGCGTGCAGCTCGCCGACCACTACCAGCAGAACACCCCC<br>ATCGGCGACGGCCCCGTGCTGCTGCCCGACAACCACTACCTGAG<br>CACCCAGTCCGCCCTGAGCAAAGACCCCAACGAGAAGCGCGATC<br>ACATGGTCCTGCTGGAGTTCGTGACCGCCGCCGGGATCACTCTC<br>GGCATGGACGAGCTGTACAAGTAGTCCTGATAACCGCCCACAGA<br>AGC
*Tg*ApiCox25 tag fwd	GAAATCCCCTCTCTGTTCCTAGAGGCAAACTGGAGCCACCTCACT<br>TTCACGGTGGAGGTAGCGGTGGTGGAAG
*Tg*ApiCox25 tag rvs	ACACAAGGATCGCATACCATAGCACGCAACACAAACAGTCATTGT<br>TTGAGGCTTCTGTGGGCGGTTATCAGG
*Tg*ApiCox25 screen fwd	GATGAGTCGTCTGTGGTTCATTG
*Tg*ApiCox25 screen rvs	GCACTTCTTCTGAAAGTTGATACG

FLAG-*Tg*ApiCox13 gBlock	ATGGACTACAAAGACCATGACGGTGATTATAAAGATCATGACATCGATTACAAGGATGACGATGACAAGGGTGGAGGTAGCGGTGGTGGAAGTCCTAGGGTGTGGCAAGAGTGGTGGCCGTATGACCCGCAGCCGCAGCCCCAGACGACGAATCCGTATCTCGTCCACTGCGAGAAAGGGAAGGTTTACTGGTGGTGCTCCTGCGGTCTCAGCAAAACACAGCCGTGGTGCGATGGCGCCCACAAAGGCACCCCTTTCAAGCCGGTTATGTACATTCCGTCGATCACAGGAAAGAAGTTGCTGTGTGGGTGTAAGCACAGCGGCAGCCGGCCACTGTGCAACGGCACTCACTTGTGGGTGAAGTGCAACAACAACACTCCCCTTGCATGCGTAGCTTCCTTCGCTGCTGCTTTCAGCGTCGGCGTCGCCTCCACGTACCTGATGCATGGTTAA
*Tg*ApiCox13 WT comp fwd	GATCAGATCTAAAATGGACTACAAAGACCATGACGG
*Tg*ApiCox13 WT comp rvs	GATCCTGCAGTTAACCATGCATCAGGTACGTGG
C-terminal FLAG gBlock	GTGGAGGTAGCGGTGGTGGAAGTGACTACAAAGACCATGACGGTGATTATAAAGATCATGACATCGATTACAAGGATGACGATGACAAGTAGTCCTGATAACCGCCCACAGAAGC
*Tg*MPPα tag fwd	CGCACTACGAGGAGGTACGCGCTGCTCTCCGAGCAGCGGGCGTCGGCAAGGGTGGAGGTAGCGGTGGTGGAAG
*Tg*MPPα tag rvs	ATGCAGCTTTCTTCGTTTCCGAGACCTTTCCAATTCTCTCTGCGCCCTGCGCTTCTGTGGGCGGTTATCAGG
*Tg*MPPα screen fwd	TTTCTTTTTCGCTGTCCGATA
*Tg*MPPα screen rvs	GTAGACACGTTCCTTCCTCTCG
*Tg*Cox2a tag fwd	GACAGTGGTACTGGATCTACGAAGTCGAGTCGCCTGTTGACGACGAaGAGGGTGGAGGTAGCGGTGGTGGAAG
*Tg*Cox2a tag rvs	CTGCCCATTCAACGCTCGGACAGCCGTCCTTTAGGAAACGCATAGGAAGCGCTTCTGTGGGCGGTTATCAGG
*Tg*Cox2a screen fwd	CTCTTGTACATGCTCGACGAAG
*Tg*Cox2a screen rvs	AACGACTGTGATTCCAAAACCT
mNG-*Tg*ApiCox13–fwd	GACGAGCTGTACAAGGCTGCAGCAATGGTGTGGCAAGAGTGGTG
mNG-*Tg*ApiCox13–rvs	TGAGCACAACGGTGATTAATTAATTAACCATGCATCAGGTACGTG
mNG-*Tg*ApiCox13^{TM}–fwd	GACGAGCTGTACAAGGCTGCAGCAATGGTGAAGTGCAACAACAACAC
mNG-*Tg*ApiCox13$^{\Delta TM}$–rvs	TGAGCACAACGGTGATTAATTAATTAACCATGGTTGTTGCACTTCACCCACAA
H49Q fwd	ATGGCGCCCAgAAAGGCACCC
H49Q rvs	CGCACCACGGCTGTGTTTTG
H85Q fwd	ACGGCACTCAgTTGTGGGTGAAGTG
H85Q rvs	TGCACAGTGGCCGGCTGC
H49C fwd	CGATGGCGCCtgCAAAGGCACC
H49C rvs	CACCACGGCTGTGTTTTG
H85C fwd	CAACGGCACTtgCTTGTGGGTGAAGTGCAACAAC
H85C rvs	CACAGTGGCCGGCTGCCG
*Tg*SdhB 3’ CRISPR fwd	CGAGCCGAGGTTACGCAGCGGTTTTAGAGCTAGAAATAGCAAG
Ty1 gBlock	GGTGGAGGTAGCGGTGGTGGAAGTGAGGTGCATACCAATCAAGACCCTTTGGATGAAGTCCATACCAATCAAGATCCTTTGGACGAGGTCCATACGAACCAGGACCCCTTGGACGGGGCCTGATAACCGCCCACAGAAGC
*Tg*SdhB tag fwd	AGGCTTTGGAGAAGTCGCAAAAGCTCGCGAAGGACCTTGGCCTCGCTGCGGGTGGAGGTAGCGGTGGTGGAAG
*Tg*SdhB tag rvs	CCTCCCGGAGGCATGCCTAGGTCCCCACATCGACTGCTCCGAGTGTACGGGCTTCTGTGGGCGGTTATCAGG
*Tg*SdhB screen fwd	GTTGTCGCTTTGATTATCTGGTG
*Tg*SdhB screen rvs	GTCACCGAGGATACACCAACG
*Tg*mtFDX 3’ CRISPR fwd	CTCAGTGGGGCGCCGGAACAGTTTTAGAGCTAGAAATAGCAAG

*Tg*mtFDX tag fwd	CTTCCGCAGATAACGCGGAACTTTTACGTCGATGGACATGTTCCG GCGCCCCACGGTGGAGGTAGCGGTGGTGGAAG
*Tg*mtFDX tag rvs	ACAGCACCGGGCTAAACGGATCCTGTCATTAGTGAATTAATATGG CCACCGCTTCTGTGGGCGGTTATCAGG
*Tg*mtFDX screen fwd	GGTCTTAGCTACTGGGATGGTG
*Tg*mtFDX screen rvs	TTGCCATTCAGAAATAAACCCT
