# Supplementary material for: An essential role for an Fe-S cluster protein in the cytochrome c oxidase complex of Toxoplasma parasites
Source: PLoS Pathog. 2023 Jun 1;19(6):e1011430. doi: 10.1371/journal.ppat.1011430 (PMC10263302; doi:10.1371/journal.ppat.1011430)
Supplement: Attachment — Submitted filename: Response to reviewers.pdf [file ppat.1011430.s013.pdf]

We have now addressed the reviewer comments through additional experimentation, data analyses, and changes to the manuscript. We describe these responses in detail below, and have numbered the reviewer comments to enable easier cross-referencing. Reviewer text is in black and our responses are in blue. We have indicated changes to the manuscript text in a marked up manuscript (changes in red text).

Reviewer #1 Major Issues

1. My only main criticism is that while the authors provide an extensive metabolic analysis (including the elegant use of Seahorse measurements to assess the involvement of TgApiCox13 in complex IV function), they did not analyse in details the consequences of TgApiCox13 depletion at the cellular level. As mentioned by the authors themselves (l. 545-549), mETC inhibition has been linked previously to T. gondii tachyzoites initiating a differentiation to the slow-growing bradyzoite stage, which has important implications for parasite persistence, and thus for designing anti-parasitic strategies. There are two very simple experiments that could be done to assess whether or not the TgApiCox13 mutant also follows the same trend, and which I think would bring important insights into the overall impact of TgApiCox13 depletion on parasite viability.

    a. The first one would be to do an ATc washout experiment to see if the parasites can survive TgApiCox13 depletion to some extent: this can be simply done by performing a plaque assay and then removing the ATc from the mutant cell line after a week and monitoring the potential appearance of plaques over the course of the following week.

As suggested by the reviewer, we have now conducted an ATc washout experiment to see if parasites can survive rHA-*Tg*ApiCox13 depletion. We include these data as S5 Fig in the supplementary data. These data indicate a) that *Tg*ApiCox13 depletion is reversible to a similar extent as another Complex IV protein (*Tg*ApiCox25), b) that *Tg*ApiCox13 depletion is more reversible than depletion of a core protein from the mitochondrial Fe-S cluster synthesis pathway (*Tg*NFS1), and c) that *Tg*ApiCox13 depletion is less reversible than depletion of a Complex III protein (*Tg*QCR11). We interpret these data as indicating that *T*gApiCox13 (and Complex IV generally) does not have a central role in mitochondrial Fe-S cluster biogenesis (consistent with other data we have obtained in response to reviewer query 2). These data also suggest that differences exist in reversibility following the loss of Complex III vs Complex IV proteins. One possible interpretation is that Complex IV has additional functions beyond the ETC (*e.g.* in biogenesis of some Fe-S proteins), although it is equally possible that the differences in reversibility between Complexes III and IV depletion have other explanations (*e.g.* that they result from differences in the speed or ability of Complexes III and IV to reassemble). Elucidating the nature of these differences is now of particular interest, but will require considerable extra work that we believe goes beyond the scope of our study. We discuss these results on lines 275-308 and place these findings into broader context in the discussion on lines 703-708.

    b. The second experiment would be to look for the initiation of parasite differentiation upon TgApiCox13 depletion by ATc through a simple Dolichol biflorus lectin staining and microscopic observation to check for the appearance

of this cyst wall marker over the course of a few days (atovaquone may be used as a positive control).

In our hands, the parental lines of the *T. gondii* parasites that we have used in our study do not readily differentiate into bradyzoites. We therefore did not do the suggested experiment. The aim of our study was to determine the role of *Tg*ApiCox13 in the cellular biology of the parasite. We demonstrate that *Tg*ApiCox13 plays a key role in Complex IV, with loss of *Tg*ApiCox13 leading to changes in cell physiology (and parasite proliferation) consistent with a selective defect in the ETC. We agree with the reviewer that elucidating the link between inhibition of the ETC (and mitochondrial Fe-S cluster biogenesis) and differentiation into bradyzoites will be an important area of future study. We acknowledge this in the discussion (lines 657-662), although in our opinion analysing this experimentally goes beyond the scope of our particular study.

Reviewer #2: Major point

2. Can TgCox13 transfer Fe-S clusters to other proteins? A common feature of human mitoNEET proteins is to transfer Fe-S clusters to other proteins (MitoNEET/PMID: 21788481, NAF-1/PMID: 24706857, MiNT/PMID: 35135884, and many other publications). Although localization of mitoNEET proteins in Toxoplasma might be different from human counterparts, the highly conserved sequence and domain features indicate that TgCox13 can potentially transfer Fe-S. Therefore, the Fe-S cluster transfer experiment is a logically must-do experiment because this will reveal TgCox13's actual functionality. Whether TgCox13 transfers Fe-S clusters must be verified.

The reviewer raises an important question that we attempted to address through a range of additional experiments.

First, we attempted to perform spectroscopic experiments to determine whether *Tg*ApiCox13 can transfer Fe-S clusters to ferredoxin (a 'model' Fe-S protein) *in vitro*. We expressed *Tg*ApiCox13$^{1\text{-}93}$, *Tg*ApiCox13$^{H49/85C}$ and ferredoxin in *E. coli*, and attempted Fe-S cluster transfer kinetic and decay experiments using UV-vis absorption spectroscopy as described previously (PMID: 21788481, 24914968 and 24706857). We recorded UV-Vis absorption spectra from 350 to 700 nm for 6 h. We did not observe Fe-S cluster transfer in the conditions tested. Although this could indicate an inability of *Tg*ApiCox13 to transfer Fe-S clusters, we cannot rule out the possibility that *Tg*ApiCox13 might transfer Fe-S clusters under alternate conditions or to other acceptor proteins. We therefore did not include these data in our manuscript.

Since the *in vitro* experiments did not give us any clear insights into whether *Tg*ApiCox13 can transfer Fe-S clusters, we next attempted to address this further in parasites. We generated a parasite line expressing a *Tg*ApiCox13 protein with both CDGSH histidine residues mutated to cysteine (H49/85C). Based on equivalent experiments with H-to-C mutations in the related *Hs*NAF-1 protein (PMID: 24914968)), we expect these mutations to result in tighter binding of the *Tg*ApiCox13 protein to the Fe-S clusters, hindering Fe-S cluster transfer to other proteins. We found that parasites expressing only the H49/85C-mutant version of *Tg*ApiCox13 were able consume O_2 normally, indicating that these mutations did not impair the mitochondrial ETC. We also found that the resulting parasites were able to proliferate normally,

indicating that, if these mutations really do render *Tg*ApiCox13 incapable of transferring Fe-S clusters, Fe-S cluster transfer is not an essential function of *Tg*ApiCox13 in *T. gondii* parasites. These experiments are described in Fig 11 and in the results on lines 594-617.

Finally, we tested whether *Tg*ApiCox13 knockdown led to any defects in the biogenesis/abundance of select mitochondrial Fe-S proteins. We have shown previously that knockdown of a key protein of the mitochondrial Fe-S cluster synthesis pathway *Tg*NFS1 leads to a decreased abundance of the mitochondrial Fe-S protein *Tg*SdhB, which we interpreted as resulting from defects in Fe-S cluster biogenesis. Previous studies have found that human MiNT is able to transfer Fe-S clusters to ferredoxin (PMID: 29259115), another mitochondrial Fe-S cluster-containing protein. We therefore tested whether the abundances of *Tg*SdhB and the mitochondrial ferredoxin protein *Tg*mtFDX changed upon the knockdown of *Tg*ApiCox13, and, as a control, upon the knockdown of *Tg*NFS1. We found that, as expected, *Tg*NFS1 knockdown led to a significant depletion of both *Tg*SdhB and *Tg*mtFDX. By contrast, *Tg*ApiCox13 knockdown did not result in a changed abundance of *Tg*SdhB, and a small (~30%) decrease in the abundance of *Tg*mtFDX (Fig 10). These data point to a minimal role for *Tg*ApiCox13 in the biogenesis of the tested mitochondrial Fe-S proteins. These experiments are described in Fig 10 and in the results on lines 574-593.

Taken together, the three additional experiments that we have undertaken suggest, at best, a minimal role for *Tg*ApiCox13 in general Fe-S transfer processes and Fe-S protein biogenesis in the mitochondrion, with its role in Complex IV being its primary function. However, our data do not rule out a more selective role for *Tg*ApiCox13 in the biogenesis of some other Fe-S cluster proteins. One possibility (as the reviewer raises in query 3) is based on recent studies that imply a role for human MiNT in transferring mitochondrial Fe-S clusters to the cytosolic Fe-S cluster assembly pathway (PMID: 35135884). This raises the possibility that *Tg*ApiCox13 may have a role in the biogenesis of cytosolic Fe-S proteins, although we have no evidence to support or refute this possibility (discussed in lines 691-708). Understanding the connections between mitochondrial and cytosolic Fe-S proteins and synthesis pathways will be an interesting area for future study, although in our opinion is beyond the scope of the present manuscript.

3. If TgCox13 can transfer Fe-S clusters, at least two hypotheses could be generated. One is TgCox13's role in delivering Fe-S clusters to other ETC complexes such as Complex II and Complex III. Formation of super-complexes of ETC complexes supports this hypothesis. The second hypothesis is TgCox13's role in transferring Fe-S clusters made in the mitochondrion to the cytosol. The authors have shown that TgCox13 relies on the mitochondrial iron-sulfur cluster (ISC) pathway. Therefore, the likelihood of exporting Fe-S by TgCox13 cannot be ruled out.

Our data are not consistent with the first hypothesis. Complex III remains intact upon *Tg*ApiCox13 knockdown (Fig 5C), and the Fe-S protein of Complex II (*Tg*SdhB) does not change in abundance (Fig 10A-B), arguing against a role for *Tg*ApiCox13 delivering Fe-S clusters to Complexes II or III. As for the second hypothesis, the idea that *Tg*ApiCox13 could be involved in Fe-S cluster export is interesting. See our

response to query 2 – we now discuss this possibility in lines 691-708 of the discussion.

4. On the other hand, if TgCox13 cannot transfer Fe-S clusters to other proteins (which will be a surprise), then it is likely that TgCox13 performs its function via oxidation-reduction of the iron and may directly transfer electrons within the Complex IV itself.

As noted in our response to query 2, we cannot conclude definitively from our results whether or not *Tg*ApiCox13 transfers Fe-S clusters. We discuss the likelihood of whether *Tg*ApiCox13 transfers electrons within Complex IV in the discussion on lines 709-735 with reference to the recent *Tetrahymena* structure (S10 Fig). Based on our analyses, the distances between the Fe-S clusters of COXFS in *Tetrahymena* and the other electron transfer sites in Complex IV are probably too large to enable efficient electron transfer within the complex.

5. In Discussion, the authors should have a short summary of NEET proteins' Fe-S cluster transfer ability and their roles in mitochondrial physiology in organisms studied so far (mostly model systems for sure). This will help emphasizing the unique role of this conserved protein in protozoan such as Toxoplasma.

We have now included a section in the introduction talking about the NEET protein family and their roles on lines 88-99. We have also included mention of this in the discussion lines 676-677 and 691-700.

Reviewer #3 Major points

6. Fig 8A-C and Fig 9A. depletion of the NFS1 protein involved in Fe-S cluster synthesis reduced the protein abundance of TgApiCox13 and the authors explained this as the Fe-S binding was important to the stability of TgApiCox13. As such, when the point mutations (H49Q and/ or H85Q) were used to complement the TgApiCox13 depletion strain, the protein levels would have been different because these different mutant alleles had different Fe-S binding capacity. Such information could not be identified without a loading control in Fig 8A-C, but was available in Fig 9A. however, I am puzzled by the protein abundance patter presented here. Can the authors explain why the abundance of FLAG-TgApiCox13 of H49Q was different in parasites treated with and without ATc? And such difference was not seen with the H49Q/H85Q mutant?

The data in Fig 7F-G indicate that mitochondrial Fe-S cluster synthesis is important for *Tg*ApiCox13 stability (as we and others have observed for the stability of other mitochondrial Fe-S proteins (PMID: 33222310 and 34793583)). Loss of Fe-S cluster synthesis would be expected to result in a decrease in the biogenesis of both Fe-S clusters of *Tg*ApiCox13, and we do indeed observe that the "H49/85Q" protein (which is defective in Fe-S cluster binding at both sites) is less abundant when expressed from the same a constitutive promoter as wild type *Tg*ApiCox13 and the single H48Q and H85Q mutants (Fig 9A). We speculate in lines 547-551 that that the observed *increase* in abundances of the H49Q and H85Q proteins in Complex IV upon knockdown of ATc-regulatable *Tg*ApiCox13 (Fig 9C-D) may represent a "quality control mechanism", by which the parasite preferentially integrates the most

active/stable protein into Complex IV when it is available (in this case, WT-ApiCox13) over less stable constructs (such as the single point variants). When the WT protein is not available (in the presence of ATc), the parasite may be able to compensate for the loss by integrating more of the less stable single point variants (therefore the abundances of these proteins increase in the presence of ATc). This could arise, for example, if integration of a less stable *Tg*ApiCox13 protein into Complex IV occurs more slowly than a more stable form. As the H49/85Q variant is the least stable and does not complement at all, we speculate that the parasite does not upregulate this protein as observed for the single point variants, since it is unable to integrate it into Complex IV.

7. Similarly, why the level of HA-TgApiCox13 was higher in all three mutants than in WT complements (judging from the gel)?

This can be explained by the same 'quality control' mechanism we propose in our answer to query 6. In the "cWT" parasite strain cultured in the absence of ATc, parasites are expressing both ATc-regulatable *Tg*ApiCox13 and a 'complementing' wild type copy of *Tg*ApiCox13 (expressed from a constitutive promoter). Both forms of *Tg*ApiCox13 are expected to be equally stable, and there is no preference for which integrates into the complex (Fig 9). By contrast, the H49Q and H85Q proteins are less stable than wild type *Tg*ApiCox13, meaning that parasites preferentially integrate ATc-regulatable *Tg*ApiCox13 into Complex IV when cultured in the absence of ATc (Fig 9). We discuss this in lines 547-551 of the text.

8. In addition, are the different complementation lines in Fig 8A-C isogenic (meaning that they were expressed from the same locus of the genome and driven by the same promoter)?

All constructs from the complementation experiments were expressed from tubulin promoter. We linearised the plasmids in a flanking region designed to promote homologous integration into the (non-essential) UPRT locus of the parasite, using a "Δ*ku80*" strain in which homologous integration is strongly preferred, although we did not verify that integration into the UPRT locus had actually occurred. We now note this in the methods (lines 835-836).

9. it may not be essential for this work, did the authors try to complement the TgApiCox13 depletion mutant with human MiNT or the dinoflagellate or ciliate homolog of TgApiCox13 to see whether they could complement? They may have similar functions although they localized to the mitochondria by different mechanisms (N-terminal peptide vs C-terminal TM domain).

Yes, we tried to complement the rHA-*Tg*ApiCox13 line with various *Hs*MiNT constructs.

a) FLAG-MiNT-TM: We fused the C-terminal transmembrane domain of *Tg*ApiCox13 (TM) to the human MiNT protein, and also included an N-terminal FLAG tag to enable protein detection. We expressed this construct in rHA-*Tg*ApiCox13 parasites. The resulting protein targeted to the mitochondrion, but compared to expression of wild type FLAG-*Tg*ApiCox13

(Fig Ai below) was expressed at very low levels (Figure A ii), precluding meaningful further experimentation with this strain;

b) mTP-FLAG-MiNT: We tried expressing human MiNT in in rHA-*Tg*ApiCox13 parasites. To facilitate mitochondrial targeting in *T. gondii*, we replaced the putative mitochondrial targeting signal of human MiNT with the mitochondrial targeting signal of *Tg*Hsp60, and included a FLAG tag to enable protein detection. The resulting protein was undetectable by immunofluorescence assays (Fig Aiii below), precluding meaningful further experimentation with this strain;

c) mTP-FLAG-MiNT-TM: In an attempt to improve protein stability of the FLAG-MiNT-TM construct described above, we fused this to the mitochondrial targeting signal of *Tg*Hsp60, and expressed this in rHA-*Tg*ApiCox13 parasites. We detected the resulting protein in the parasite mitochondrion by immunofluorescence assay (Fig A iv), and could detect this protein by western blotting (Fig B below), albeit at much lower abundance that the wild type FLAG-*Tg*ApiCox13 expressed from the same promoter (Fig B; note that both blots are from the same membrane and same exposure time). We performed BN-PAGE, and, in contrast to the wild type FLAG-*Tg*ApiCox13 protein, were unable to detect the resulting mTP-FLAG-MiNT-TM protein in a protein complex (Fig C below; both blots are from the same membrane and same exposure time). We also tested whether expression of the mTP-FLAG-MiNT-TM in rHA-*Tg*ApiCox13 parasites could complement parasite proliferation upon HA-*Tg*ApiCox13 knockdown, and found that it couldn’t (Fig D below).

In sum, the complementation experiments with human MiNT were inconclusive. Although we did not observe any complementation where we tested this, this could be because the resulting proteins had low abundance. We therefore chose not to include these data in the manuscript.

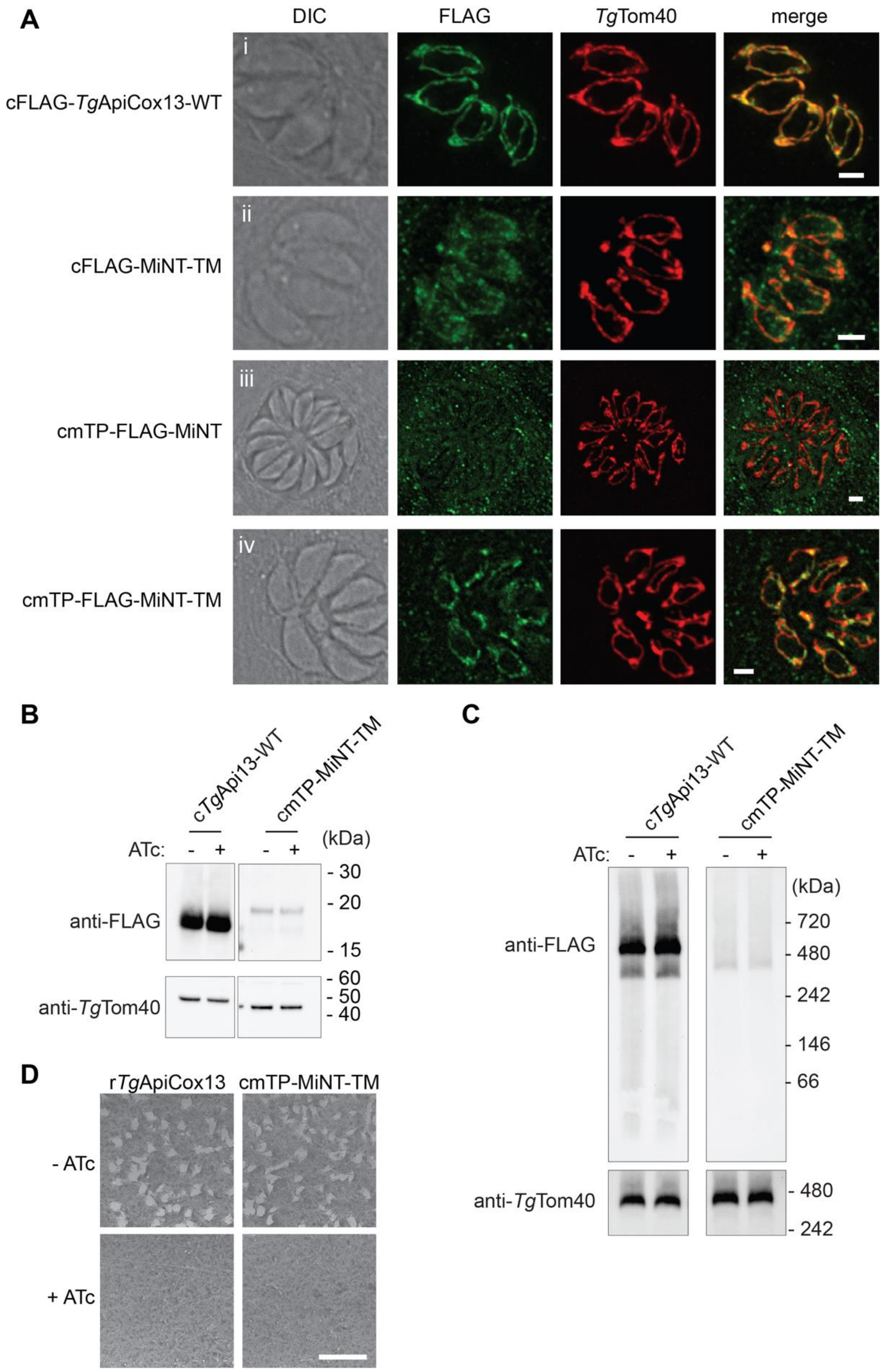
A
DIC
FLAG
TgTom40
merge
i
cFLAG-TgApiCox13-WT
ii
cFLAG-MiNT-TM
iii
cmTP-FLAG-MiNT
iv
cmTP-FLAG-MiNT-TM
B
cTgApi13-WT
cmTP-MiNT-TM
ATc:
- + - +
(kDa)
- 30
- 20
- 15
anti-FLAG
- 60
- 50
- 40
anti-TgTom40
C
cTgApi13-WT
cmTP-MiNT-TM
ATc:
- + - +
(kDa)
720
480
242
146
66
anti-FLAG
- 480
- 242
anti-TgTom40
D
rTgApiCox13
cmTP-MiNT-TM
- ATc
+ ATc

Reviewer #1 Minor points

10. L. 66: correct to “Apicomplexa is a phylum comprising intracellular parasites…” or “Apicomplexans belong to a phylum of intracellular parasites…”.

We have now changed line 70 to “Apicomplexans belong to a phylum of intracellular protozoan parasites…”.

11. Table 1: I would order this by coupling it to a phylogenetic analysis, hierarchical clustering might reveal if the C-terminal feature was inherited in related eukaryotes.

We have mapped the presence of C-terminal transmembrane domains (TMD) and N-terminal mitochondrial targeting peptides (mTP) from MiNT/ApiCox13 homologs onto an illustrative phylogenetic tree of eukaryotic evolution, which we now include as S1 Fig. Given its presence in all analysed alveolates, these data suggest that the common ancestor of alveolates had an ApiCox13 homolog with a C-terminal TMD. We have highlighted this point in the main text (lines 142-144). This tree also suggests that N-terminal mTPs on ApiCox13/MiNT homologs may have evolved multiple times across evolution. Given its broad distribution across eukaryotic taxa, it is conceivable that the last eukaryotic common ancestor had an ApiCox13 homolog with a C-terminal TMD, although this would require multiple losses in lineages such as metazoans and trypanosomatids. Alternatively, it is conceivable that the C-terminal TMD of ApiCox13/MiNT evolved multiple times across eukaryotic evolution, or that the gene encoding this protein spread through lateral gene transfer events. Given that these evolutionary possibilities are speculative, we’ve chosen not to mention this in the text, although it will be interesting in future to determine whether the TMD-containing ApiCox13/MiNT homolog in amoebozoans and chytrid fungi associates with Complex IV.

12. Figure 1. I think that adding an AlphaFold model of TgApiCox13, highlighting the two cluster-binding domains, the C-terminal helix, and perhaps also highlighting the mutated residues, would be particularly informative.

We have now included a model of *Tg*ApiCox13 structure predicted by AlphaFold as Fig 1B, highlighting the C-terminal helix and the histidine residues important for Fe-S cluster coordination. This is discussed in the results section in lines 147-155.

13. L. 244-248. Can it be speculated that the increase in ECAR might also reflect a compensatory mechanism in response to the decrease in mETC function?

We have now added a sentence to address this (lines 270-273): “We speculate that the slight increase in ECAR, which we have also observed upon the depletion of other ETC proteins (PMID: 30204084 and 33524071), may reflect a compensatory mechanism, perhaps increased glycolytic flux, in response to decreased ETC function.”

14. Fig. 4D: given the extremely high error bars displayed on the graph, I doubt this can be interpreted correctly. These data are rather accessory and might be removed.

Yes, we agree that Fig 4B illustrates this point sufficiently and have removed 4D. We instead replace it with the quantification of TMPD-elicited OCR, which was a supplementary figure.

15. Fig. 6B, C. Overexposed immunofluorescence signals, pictures should be replaced if possible.

We have now replaced Fig 6C with less exposed images. Unfortunately, we were not able to replace Fig 6B with a less-exposed image. We think that the mNG image presented in 6B does show the mitochondrial labelling quite clearly.

16. L. 392-393: the authors conclude that FeS biogenesis is essential for Complex IV activity as if it is a specific phenotype, but I wonder if the emphasis should be put on that as Fig. 7B shows that TgNFS1 mutants respond poorly to malate, ATV or TMPD, which is consistent with other FeS proteins being involved in complexes II and III also.

We are not claiming that only Complex IV is affected upon loss of *Tg*NFS1. It is indeed likely that other Complexes are affected (PMID: 33222310). However, our assay does tell us there is a defect downstream of CytC (*i.e.* in Complex IV), leading us to conclude that Fe-S cluster biogenesis is also important for Complex IV function. We changed the text to avoid any confusion on this point:

A) We added the following sentence to lines 430-434: "We have previously shown that the O_2 consumption of intact parasites is compromised upon knockdown of *Tg*NFS1 (PMID: 33222310), which is unsurprising given the importance of Fe-S cluster biogenesis for ETC Complexes II and III. To assess the functionality of ETC Complex IV, we…"
B) We removed the word "specifically" from line 446 when referring to Complex IV activity.

17. Fig. 9A, left bottom graph. I doubt that there would be no statistical significance when comparing cWT and cH49/85Q –ATc conditions: check again, or perhaps the bars are badly positioned… Also, any comment as to why there is a decrease of TgApiCox13 even in the absence of ATc?

We now report the numerical *p*-value comparing the abundances of the cWT and cH49/85Q proteins in the –ATc condition (0.055), as well as several other relevant *p*-values.

As for the decreased abundance of HA-*Tg*ApiCox13 in the -ATc condition in the parasites that also express cWT, see our response to queries 6 and 7.

18. L. 566: "a recent structural analysis of Complex IV in the ciliate Tetrahymena…"; add corresponding reference.

The corresponding references have now been added to lines 713 and 715.

Reviewer #2: Minor points

19. Table 1, it is helpful to list gene IDs that are shown in vEupthDB. That will help parasitologists to search the genes.

Table 1 has now been moved to the supplementary as S1 Table. We have added the vEuPathDB ID after the NCBI ID with a slash (/) for relevant organisms (*Plasmodium falciparum*, *Babesia bovis*, *Trypanosoma cruzi* and *Vitrella brassicaformis*).

20. Fig 2 intends to show that TgCox13 and TgCox25 belong to the same complex, as predicted by structural studies in Tetrahymena or complexome profiling in Plasmodium. The authors should also run the co-immunoprecipitation product on BN-PAGE to show that Cox13 is present in a large complex in the eluted material of Cox25 IP, and vice versa. Fig2C/2D have not provided direct evidence that TgCox13 and TgCox25 are in the same complex.

The experiment that the reviewer proposes is not possible, since we elute immunoprecipitated proteins from beads using conditions that will denature protein complexes (incubation in SDS-PAGE sample buffer or low pH glycine buffer). Our study provides the following evidence that *Tg*ApiCox13 is a *bona fide* component of Complex IV in *T. gondii*:

1. *Tg*ApiCox13 and *Tg*ApiCox25 are part of similarly sized protein complexes in BN-PAGE experiments (Fig 2C);
2. Knockdown of *Tg*ApiCox25 leads to a change in mass of the *Tg*ApiCox13 complexes, which you would expect if these proteins are part of the same complex (Fig 2C);
3. In a similar vein, knockdown of *Tg*ApiCox13 leads to a depletion of the complexes in which *Tg*ApiCox25 and *Tg*Cox2a reside (but not the TOM complex or Complex III; Fig 5A-C);
4. Immunoprecipitation of *Tg*ApiCox13 co-purified *Tg*ApiCox25 and vice versa, but not the unrelated mitochondrial protein *Tg*Tom40 (Fig 2D). This provides *direct evidence* that the two proteins associate with each other. It is highly unlikely but conceivable that they both associate with each other but are not part of the same complex. However, we find that immunoprecipitation of *Tg*ApiCox13 purifies virtually all of the *Tg*ApiCox25 protein and vice versa (Fig 2D), which would only be possible if the ~600 kDa protein complex in which both reside is in fact the same complex. Nevertheless, we have made a slight modification to the text to now read that the co-immunoprecipitation experiments provides a “more direct” test for whether ApiCox13 is a Complex IV protein than the BN-PAGE/knockdown experiments described in Figure 2C (line 225).

In addition to the evidence we provided in this study, several other lines of indirect evidence support that *Tg*ApiCox13 is a component of Complex IV in *T. gondii*. We initially identified *Tg*ApiCox13 as a candidate Complex IV protein in two proteomic studies of purified Complex IV (PMID: 30204084 and 33524071). Complexome studies of ETC complexes from *T. gondii* also identified *Tg*ApiCox13 as co-eluting with other Complex IV protein (PMID: 33651838). As the reviewer notes, homologues of *Tg*ApiCox13 were identified as a Complex IV protein in structural studies of the *Tetrahymena* respiratory chain complexes, and as co-eluting with Complex IV in a

complexome study in *Plasmodium falciparum*. We cite all of these studies in the Introduction.

21. Fig 4B/7B. Could the authors explain why 10 mM NaN3 seems not to inhibit OCR robustly (not even as good as 1 uM atovaquone)?

We have seen this consistently in previous studies (PMID: 33524071 and 35118179; Hayward et al., 2022, BioRxiv 2022.02.13.480284). We are not sure why NaN_3 does not fully inhibit OCR. It is conceivable that the divergence of *T. gondii* Complex IV may have altered the complex in some way that reduces the degree of NaN_3 inhibition. One possibility is that the *T. gondii* Complex IV NaN_3 binding site has been altered; however, given that NaN_3 binds to the catalytic heme a3 and CuB (PMID: 9624044), this explanation does not seem likely. It could also be accessibility of NaN_3 to its binding site is compromised by Complex IV divergence. For the purposes of our experiments, it still does act as a control for chemical inhibition (albeit incomplete) of Complex IV to compare to our genetic Complex IV mutants.

22. Fig 4B, data of the complemented lines shown in 4C are missing.

We chose not to include the complemented lines in Fig 4B for clarity (there were many lines overlapping each other, which made it difficult to distinguish between the different experimental treatments). As the reviewer notes, we quantify these date in Fig 4C. We have provided the raw trace data for all three repeats below.

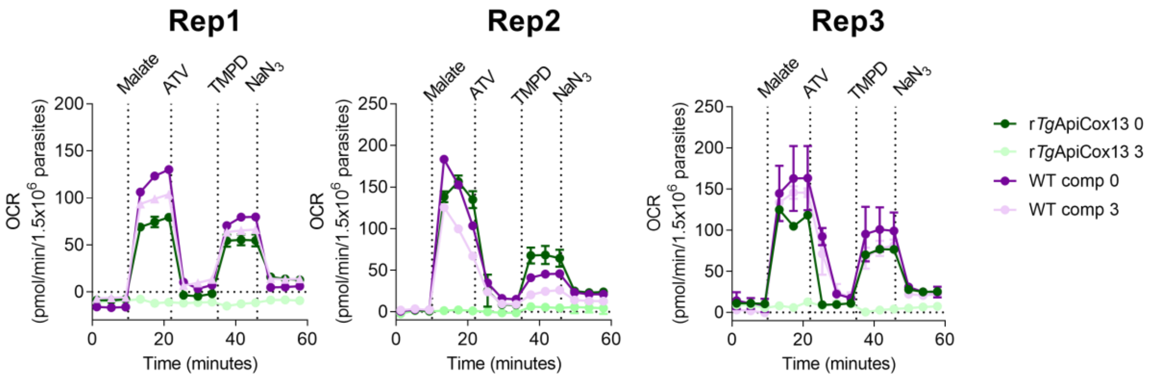

23. Fig 4E, a protein loading control is missing.

Unfortunately providing a loading control for this experiment is not technically feasible. The gel shown in 4E is an in-gel activity assay where a coloured product is produced by Complex IV. We tried to stain the gel post-assay with Coomassie to provide a total protein loading control, but found that this is not compatible with the chemistry of the post-assay gel (the gel stained completely blue). We do state in the figure legend that these experiments were performed on samples that were extracted from an equal number of parasites.

24. Fig 6A, it is hard to conclude that Cox13 is enriched in the pellet based on the data shown here. Perhaps other methods of mitochondrial membrane isolation should be used to address this issue. This is critical to interrogate the role of

TgCox13 because the matrix TgCox13 could be involved in other functions, not in Complex IV.

As noted in our response to query 20, our co-immunoprecipitation experiments (Fig 2D) demonstrate that when we immunoprecipitate *Tg*ApiCox25, almost all of *Tg*ApiCox13 is co-immunoprecipitated and vice versa. This result implies that all of *Tg*ApiCox13 is associated with Complex IV in the inner mitochondrial membrane and that there is unlikely to be free *Tg*ApiCox13 protein in the matrix.

Sodium carbonate extraction is quite dependent on pH and on the hydrophobicity of the target proteins, with proteins possessing fewer hydrophobic transmembrane domains being extracted/solubilised differently to multi-pass transmembrane proteins (PMID: 26435163). Since *Tg*ApiCox13 is predicted to have a single transmembrane helix (Fig 1A-B) we suspect that it may be more susceptible to extraction compared to the control, the highly integrated beta-barrel protein *Tg*Tom40. This would explain why we observed that while *Tg*ApiCox13 is enriched in the pellet relative to the supernatant (Fig 6A), it is present in both fractions whereas *Tg*Tom40 is solely in the pellet. We address the point in lines 395-398.

25. Fig 9A/9B, a loading control is missing for the western blots on the right panel.

The blots presented in left and right panels of Fig 9A, and the left and right panels of Fig 9B in the original manuscript were all from the same experiment (*i.e.* extracted at the same time and separated on the same gel). The *Tg*Tom40 blot presented on the right of Fig 9A and 9B therefore also acts as the loading control for the panels on the left. To prevent any confusion on this point, we re-arranged Figure 9 such that the 4 SDS-PAGE blots are arranged on top of each other (Fig 9A) with the quantification shown below (Fig 9B) and the 4 BN-PAGE blots are together (Fig 9C) with the quantification below (Fig 9D). We also make clear in the figure legend that these are matched samples from the same experiment.

26. Fig 9A, Line 468, please explain what do these up or down changes in protein abundances mean or imply.

See our response to queries 6 and 7.

Other points

27. Lines 38, remove the extra that done
28. Line 153, H is missing in 49C. changed as suggested
29. Line 244-245, could the author explain why ECAR is increased? See response to query 13
30. Line 423, Line 426, Line 439, 84Q should be 85Q changed as suggested
31. Line 605, what type of French press? Now stated (lines 750-751)
32. Line 631/857/858, ug should be µg, uL should be µL changed as suggested
33. Lines 689/698/700/715/724/727/737 and also the main text, strains should be lines. changed as suggested
34. Line 881, should distance be bold? We have now changed “distance” to be in quotations to show that it is the name of a command in Chimera.

Reviewer #3:

35. table 1 is somewhat redundant with figure 1. It may be placed to the supplemental files. We agree, and have now moved Table 1 from the main text to the supplementary (now S1 Table).
36. line 271, the full name of TMPD is needed. We added the full name N,N,N',N'-tetramethyl-p-phenylenediamine dihydrochloride to line 331-332.
37. lines 423, 426 and 439 (likely elsewhere as well), should be "cH49/85Q" changed as suggested
38. Fig 4E, it would be helpful to indicate what exactly the readers are looking? What does that band mean? We say both in the Fig 4E label beneath the gel and in the figure legend that this is showing Complex IV activity. We have added an additional line to the figure legend to specify that band intensity corresponds to Complex IV activity (line 1379-1380). *i.e.* A decrease in staining/coloured product on the gel indicates decreased Complex IV activity. We show later (Fig 5) that this is due to decrease in the abundance of Complex IV.
39. line 602, expression commonly requires a lysogenic DE3 phage, so the strain is likely BL21(DE3), please check. Yes, we have now changed this to BL21(DE3)
40. line 636, "an HA tag" changed as suggested
